# Automated Pupillometry for Prediction of Electroencephalographic Reactivity in Critically Ill Patients: A Prospective Cohort Study

**DOI:** 10.3389/fneur.2022.867603

**Published:** 2022-03-21

**Authors:** Lorenzo Peluso, Lorenzo Ferlini, Marta Talamonti, Narcisse Ndieugnou Djangang, Elisa Gouvea Bogossian, Marco Menozzi, Filippo Annoni, Elisabetta Macchini, Benjamin Legros, Paolo Severgnini, Jacques Creteur, Mauro Oddo, Jean-Louis Vincent, Nicolas Gaspard, Fabio Silvio Taccone

**Affiliations:** ^1^Department of Intensive Care, Erasme University Hospital, Brussels, Belgium; ^2^Department of Neurology, Erasme University Hospital, Brussels, Belgium; ^3^Department of Biotechnology and Life Sciences, Insubria University, Cardiac Anesthesiology and Intensive Care - ASST Sette Laghi, Varese, Italy; ^4^Critical Care Clinical Research Unit, Department of Intensive Care Medicine, CHUV-Lausanne University Hospital, Lausanne, Switzerland; ^5^Department of Neurology, Yale University Medical School, New Haven, CT, United States

**Keywords:** brain injury, encephalopathy, intensive care unit, autonomic dysfunction, EEG, pupillary function

## Abstract

**Background:**

Electroencephalography (EEG) is widely used to monitor critically ill patients. However, EEG interpretation requires the presence of an experienced neurophysiologist and is time-consuming. Aim of this study was to evaluate whether parameters derived from an automated pupillometer (AP) might help to assess the degree of cerebral dysfunction in critically ill patients.

**Methods:**

Prospective study conducted in the Department of Intensive Care of Erasme University Hospital in Brussels, Belgium. Pupillary assessments were performed using the AP in three subgroups of patients, concomitantly monitored with continuous EEG: “anoxic brain injury”, “Non-anoxic brain injury” and “other diseases”. An independent neurologist blinded to patient's history and AP results scored the degree of encephalopathy and reactivity on EEG using a standardized scale. The mean value of Neurologic Pupil Index (NPi), pupillary size, constriction rate, constriction and dilation velocity (CV and DV) and latency for both eyes, obtained using the NPi®-200 (Neuroptics, Laguna Hills, CA, USA), were reported.

**Results:**

We included 214 patients (mean age 60 years, 55% male). EEG tracings were categorized as: mild (*n* = 111, 52%), moderate (*n* = 65, 30%) or severe (*n* = 16, 8%) encephalopathy; burst-suppression (*n* = 19, 9%) or suppression background (*n* = 3, 1%); a total of 38 (18%) EEG were classified as “unreactive”. We found a significant difference in all pupillometry variables among different EEG categories. Moreover, an unreactive EEG was associated with lower NPi, pupil size, pupillary reactivity, CV and DV and a higher latency than reactive recordings. Low DV (Odds ratio 0.020 [95% confidence intervals 0.002–0.163]; *p* < 0.01) was independently associated with an unreactive EEG, together with the use of analgesic/sedative drugs and high lactate concentrations. In particular, DV values had an area under the curve (AUC) of 0.86 [0.79–0.92; *p* < 0.01] to predict the presence of unreactive EEG. In subgroups analyses, AUC of DV to predict unreactive EEG was lower (0.72 [0.56–0.87]; *p* < 0.01) in anoxic brain injury than Non-anoxic brain injury (0.92 [0.85–1.00]; *p* < 0.01) and other diseases (0.96 [0.90–1.00]; *p* < 0.01).

**Conclusions:**

This study suggests that low DV measured by the AP might effectively identify an unreactive EEG background, in particular in critically ill patients without anoxic brain injury.

## Introduction

Alterations of consciousness and neurological injury are associated with high morbidity and mortality in critically ill patients, independent of the nature of the brain damage (1). Neuromonitoring is essential to detect changes in cerebral function, to identify the underlying pathological process(es), and to guide interventions aimed at preventing secondary brain injury (2, 3). Clinical examination can be unreliable when sedatives are used, when there are systemic complications, or if the initial brain injury is severe (4), so that additional monitoring tools are required in this setting (5).

Continuous electroencephalography (cEEG) is one of the most widely used tools in the monitoring of acute neurological conditions; clinically relevant findings from electroencephalography include not only the presence of seizures or status epilepticus, but also the background and reactivity of the electroencephalogram (EEG) (e.g., to assess the severity of encephalopathy, as in Post-anoxic brain injury or sepsis) (6, 7). As EEG interpretation requires an experienced neurophysiologist and, for some analyses, remains time- and resource-consuming (8), it would be interesting to know whether other invasive or Non-invasive neuromonitoring tools correlate with some of the main EEG findings and could help identify patients who should be considered for electroencephalograph monitoring. Among those tools, quantitative automated pupillometry has recently been introduced into clinical practice and its utility and reliability to monitor patients with different neurological conditions have been well described (3, 9, 10). Indeed, automated pupillometry not only provides a quantitative and reliable assessment of pupillary size and degree of constriction or dilation to light or painful stimuli, but also information on brainstem integrity, cortical activity, and autonomic nervous system function (11).

A pilot study showed that the reduction in pupillary constriction rate to light stimulation derived from automated pupillometry identified patients “at risk” of an unreactive background EEG (12). However, this study had several limitations, including the small sample size, lack of adjustment for several confounders, e.g., use of sedatives or underlying brain injury, and lack of additional automated pupillometry-derived variables, such as the Neurological Pupil index (NPi), which has been shown to predict neurological outcome in critically ill patients (13, 14).

The aim of this study was therefore to investigate the correlation between EEG reactivity and parameters derived from automated pupillometry in a larger sample of ICU patients and to determine the independent predictive value of automated pupillometry-derived variables for an unreactive EEG. We also evaluated whether the underlying brain condition could influence these associations.

## Methods

### Study Design and Patient Selection

This prospective observational study was performed in the Department of Intensive Care (ICU) at Erasme Hospital, Brussels (Belgium) between October 15, 2018 and December 17, 2019. Eligible patients were those requiring a cEEG, according to the decision of the attending physician (i.e., assessment of prognosis for anoxic brain injury, detection of seizures and asymmetries in Non-anoxic brain injury and detection of Non-convulsive seizures or excessive sedation in other diseases), and in whom pupillary assessment was performed using an automated pupillometry (NPi®-200 pupillometer; Neuroptics, Laguna Hills, CA, USA-eMethods1 in the Supplement), as for routine practice. The local Ethical Committee (Comité d'Ethique Hospitalo-Facultaire Erasme-ULB) approved the study protocol (P2018/308), but waived the need for informed consent because cEEG and pupillometry are both standard of care in patients with brain dysfunction or altered level of consciousness in our department. The design and methodology of the study is in accordance with STROBE (STrengthening the Reporting of OBservational studies in Epidemiology) for cohort study (Supplementary Methods 1).

### EEG Recording and Definition

Twenty-one electroencephalograph electrodes were placed on the scalp, according to the International 10–20 system and cEEGs were recorded using a clinical grade system (BrainRT, OSG Inc., Rumst, Belgium). A 20-min epoch around the onset of the automated pupillometry assessment was assessed according to the definitions of American Clinical Neurophysiology Society's Standardized Critical Care EEG Terminology (15) by a neurophysiologist (LF), who was not involved in the care of the patients and was unaware of their diagnoses and of the results of the pupillometry. The EEG background was classified into one of five categories of encephalopathy using a modified Synek scale (Supplementary Table 1). The presence of EEG changes to external stimulation (i.e., name call for awake and painful stimuli for unconscious patients), defined as “EEG reactivity”, was also assessed during the same epoch, categorizing the EEG background pattern as “reactive” or “unreactive” (12). The physicians who performed the pupillometry were not involved in the care of the patients and were unable to interpret EEG features (Supplementary Methods 2).

### Definitions

Patients were categorized into three groups according to their admission diagnosis: i) “anoxic brain injury” (i.e., comatose Post-cardiac arrest patients); ii) “Non-anoxic brain injury” (i.e., subarachnoid hemorrhage [SAH]; traumatic brain injury [TBI]; haemorrhagic and ischemic stroke, central nervous system [CNS] infections); iii) “other diseases” (e.g., sepsis, liver failure, metabolic alterations, excessive sedation, etc.).

### Outcome Measures

The primary outcome of the study was to identify the variable derived from automated pupillometry that had the highest predictive value for an unreactive EEG. Secondary outcomes were: a) to identify independent predictors of unreactive EEG; b) to assess the association of the parameters derived from automated pupillometry with different degree of encephalopathy; c) to assess the correlation between pupillary parameters and clinical evaluation of consciousness (i.e., Glasgow Coma Scale [GCS]); d) to evaluate the predictive value of automated pupillometry-derived variables for an unreactive EEG within the different subgroups of brain-injured patients; e) to assess the association of automated pupillometry-derived parameters with mortality.

### Statistical Analysis

Discrete variables are expressed as count (percentage) and continuous variables as mean ± standard deviation (SD) or median [25th to 75th percentiles]. The Kolmogorov-Smirnov test was used, and histograms and normal-quantile plots were examined to verify the normality of distribution of continuous variables. Differences in demographic, clinical, and EEG patterns among groups were assessed using the chi-square test or Fisher's exact test for categorical variables, as appropriate; for continuous variables, we used a Student's *t*-test or Mann–Whitney U-test, as appropriate. For comparison of continuous variables with more than two groups we used a one-way ANOVA test or a Kruskal-Wallis test, as appropriate and performed *post-hoc* pairwise comparisons through a Bonferroni correction. Multivariable logistic regression analysis with the unreactive EEG group as the dependent variable was performed; co-linearity between continuous (i.e., a linear correlation coefficient > 0.3) and categorical (i.e., variance inflation factor > 10) variables was excluded prior to modeling; only variables associated with unreactive EEG in the univariate analysis (*p* < 0.05) were included in the multivariable model. Odds ratios (OR) with 95% confidence intervals (CI) were computed; goodness-of-fit of the model was assessed using the Hosmer and Lemeshow test. The ability of pupillometry values to predict an unreactive EEG was tested with different receiver operating characteristics (ROC) curves, and the area under the curve (AUC) for each subgroup of patients was calculated. Three thresholds for each diagnosis subgroup were calculated: the first to have a specificity >95%, the second using the threshold with the best Youden's index, and the third to have a sensitivity >95%. We compared the performance of the different ROC curves using DeLong's test. For a sensitivity analysis, we also performed the multivariable analysis in two additional subgroups: a) excluding patients with mild encephalopathy (i.e., awake with a clinical status suggestive of a reactive EEG); b) including only sedated patients. All statistical tests were two-tailed and a *p* value < 0.05 was considered as statistically significant. Data were analyzed using IBM SPSS Statistics for Macintosh 25 (Armonk, NY, USA) and GraphPad Prism 9 (San Diego, CA, USA).

## Results

### Study Population

Among 464 consecutive patients who underwent cEEG, 42 were excluded (24 because of ocular trauma or previous ocular surgery and 18 because of severe agitation with unreliable automated pupillometry assessment) and 208 were not enrolled because of the absence of the operator to record the exact time of automated pupillometry assessment on the cEEG. A total of 214 patients were therefore included in the final analysis. The characteristics of the study population are shown in Table 1 and Supplementary Table 2. Forty-five (21%) patients had anoxic brain injury, 124 (58%) had Non-anoxic brain injury and 45 (21%) other diseases; admission diagnoses are given in Supplementary Table 3.

**Table 1:** Characteristics of the study population, according to diagnostic subgroup (complete data are shown in Supplementary Table 2).

**Characteristic**	**Overall (*n* = 214)**	**Anoxic Brain Injury (*n* = 45)**	**Not-Anoxic Brain Injury (*n* = 124)**	**Other Diseases (*n* = 45)**
Age, years	60 [50–72]	68 [57–74]^a^	57 [48–70]^b^	66 [55–73]^a, b^^*^
Men, *n* (%)	117 (55)	34 (76)^a^	58 (47)^b^	25 (56)^a, b^^*^
GCS, *n*	9 [3–14]	3 [3–4]^a^	10 [7–14]^b^	10 [5–14]^b^^*^
Time from admission to test, days	2 [1–3]	1 [1–2]^a^	2 [1–4]^b^	2 [1–3]^b^^*^
**Comorbidities**				
COPD, *n* (%)	29 (14)	6 (14)	15 (12)	8 (18)
Heart disease, *n* (%)	76 (35)	25 (57)^a^	27 (22)^b^	24 (53)^a^^*^
Arterial hypertension, *n* (%)	94 (44)	22 (50)	58 (47)	14 (31)
Diabetes, *n* (%)	37 (17)	8 (18)	20 (16)	9 (20)
Liver cirrhosis, *n* (%)	15 (7)	1 (2)^a^	4 (3)^a^	10 (23)^b^^*^
Chronic renal disease, *n* (%)	33 (15)	11 (25)^a^	9 (7)^b^	13 (29)^a^^*^
Previous neurologic disease, *n* (%)	47 (22)	8 (18)	27 (22)	12 (27)
**Drugs during measurement**				
Sedatives, *n* (%)	62 (29)	34 (76)^a^	20 (16)^b+^	8 (18)^b^^*^
Opioids, *n* (%)	78 (37)	38 (84)^a^	32 (26)^b^	8 (18)^b^^*^
Analgosedation	125 (58)	4 (9)^a^	86 (69)^b^	35 (79)^b^^*^
*No Drugs, n (%)*	11 (5)	3 (7)^a^	6 (5)^a^	2 (4)^a^
*Sedatives, n (%)*	27 (13)	7 (15)^a^	18 (15)^a^	2 (4)^a^
*Opioids, n (%)*	51 (24)	31 (69)^a^	14 (11)^b^	6 (13)^b^
*Sedative and Opioids, n (%)*				
Vasopressors, *n* (%)	106 (50)	37 (82)^a^	44 (36)^b^	25 (56)^b^^*^
Inotropes, *n* (%)	25 (12)	18 (40)^a^	2 (2)^b^	5 (11)^c^^*^
**Pupillometry values**				
NPi Mean	4.6 [4.3–4.8]	4.6 [4.3–4.8]^a, b^	4.6 [4.1–4.8]^a^	4.7 [4.4–4.8]^b^^*^
Size Mean, mm	3.37 [2.45–4.35]	2.40 [2.04–3.22]^a^	3.64 [2.90–4.60]^b^	3.30 [2.39–4.22]^b^^*^
CH Mean, %	31 [20–39]	21 [14–31]^a^	33 [21–40]^b^	34 [26–41]^b^^*^
CV Mean, mm/s	1.62 [0.95–2.44]	0.98 [0.65–1.33]^a^	1.91 [1.28–2.62]^b^	1.86 [1.03–2.39]^b^^*^
MCV Mean, mm/s	2.63 [1.50–4.06]	1.46 [1.02–2.21]^a^	3.06 [1.98–4.48]^b^	3.06 [1.56–4.08]^b^^*^
LAT Mean, sec	0.25 [0.22–0.27]	0.27 [0.24–0.30]^a^	0.23 [0.22–0.27]^b^	0.26 [0.23–0.29]^a^^*^
DV Mean, mm/s	0.64 [0.37–0.96]	0.37 [0.26–0.67]^a^	0.73 [0.42–1.01]^b^	0.79 [0.47–1.02]^b^^*^
**EEG Features**				
Background categories	111 (52)	7 (16)^a^	80 (65)^b^	^*^
*Mild encephalopathy*	65 (30)	15 (33)^a^	36 (29)^a^	24 (53)^b^
*Moderate encephalopathy*	16 (8)	9 (20)^a^	3 (2)^b^	14 (31)^a^
*Severe encephalopathy*	19 (9)	12 (27)^a^	5 (4)^b^	4 (9)^a, b^
*Burst suppression*	3 (1)	2 (4)^a^	0 (0)^a^	2 (5)^b^
*Suppression*				1 (2)^a^
Unreactive EEG, *n* (%)	38 (18)	23 (51)^a^	8 (6)^b^	7 (16)^b^^*^
**Outcome Variables**				
ICU mortality, *n* (%)	70 (33)	28 (62)^a^	29 (23)^b^	13 (29)^b^^*^
Hospital mortality, *n* (%)	87 (41)	32 (71)^a^	36 (29)^b^	19 (43)^b^^*^

*Results given as count (%) or median [IQR]*.

*GCS, Glasgow Coma Scale; COPD, Chronic Obstructive Pulmonary Disease; NPi, Neurologic Pupil Index; CH, Constriction Percentage; CV, Constriction Velocity; MCV, Maximum Constriction Velocity; LAT, Latency; DV, Dilation Velocity; ICU, Intensive Care Unit*.

* *= p < 0.05. Pairwise comparison calculated using Dunn-Bonferroni correction and are expressed with superscript letters (equal letters indicate no difference among subgroups)*.

### cEEG Findings, GCS and Correlation With Pupillometry Variables

**Figure 1:**
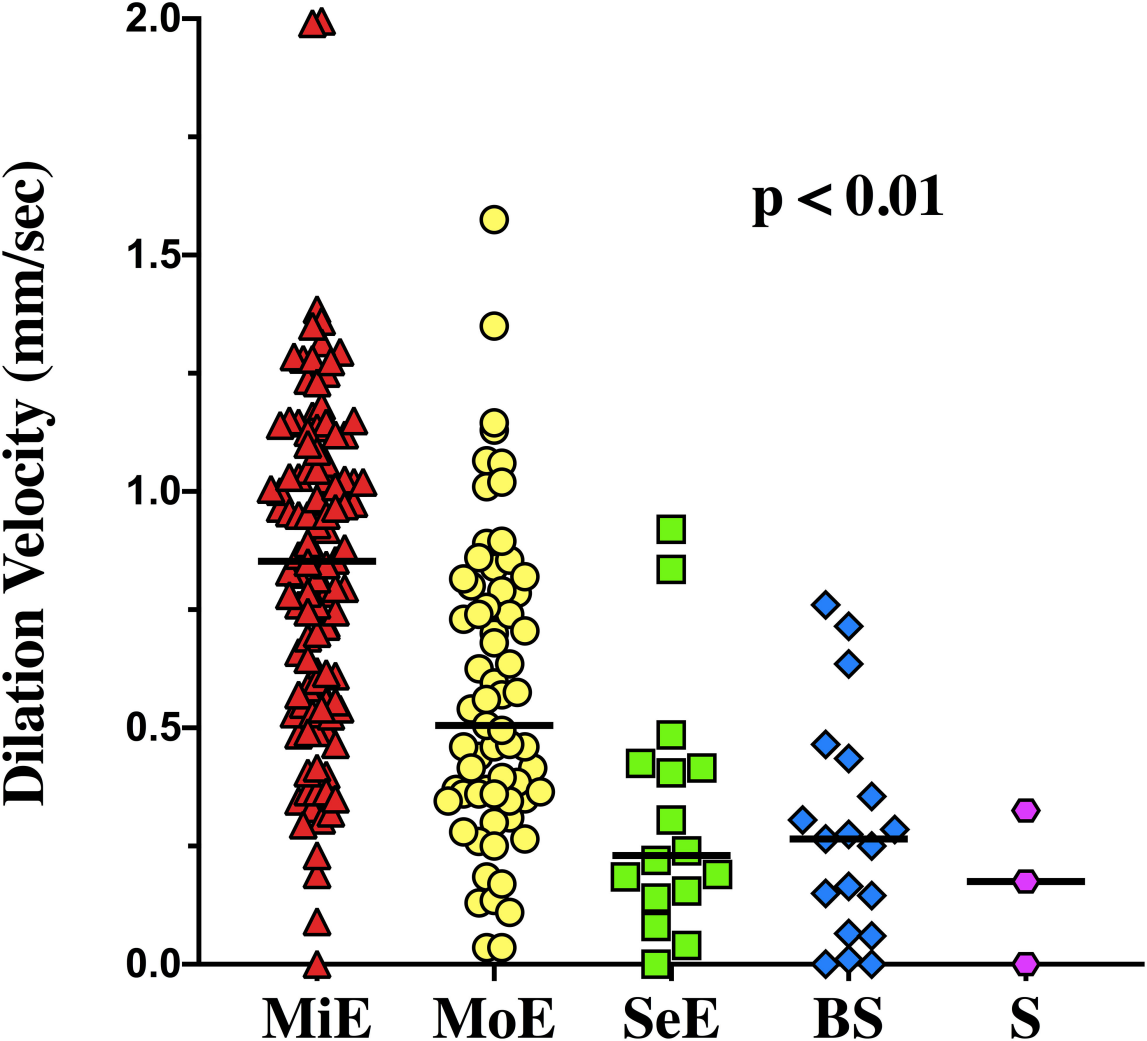
Dilation velocity values among the different categories of encephalopathy. MiE, Mild Encephalopathy, MoE, Moderate Encephalopathy, SeE, Severe Encephalopathy, BS, Burst Suppression, S, Suppression.

**Figure 2:**
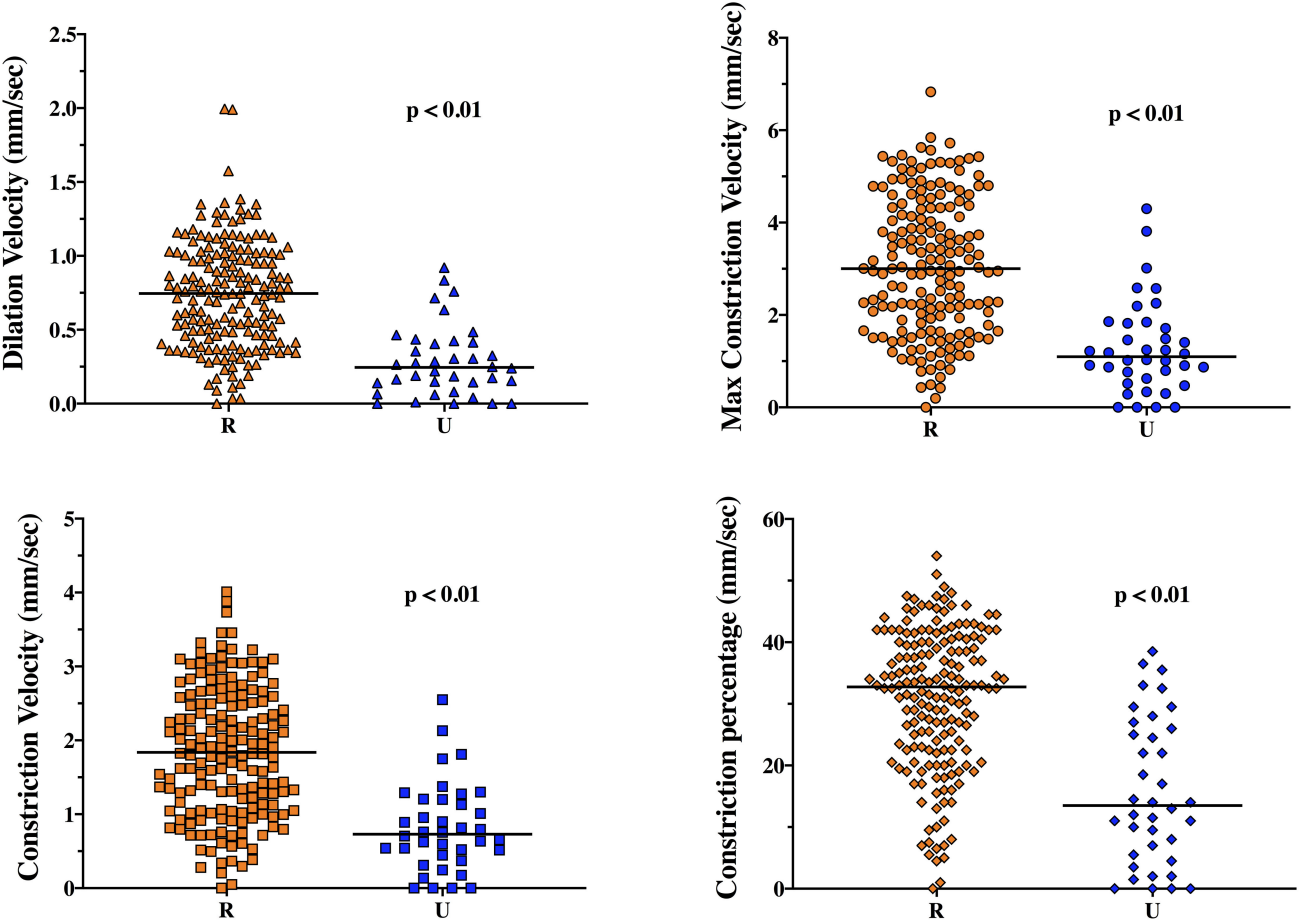
Dilation velocity (DV), maximum constriction velocity (MCV), constriction velocity (CV) and constriction percentage (CH) in reactive (R) and unreactive (U) EEG recordings.

**Figure 3:**
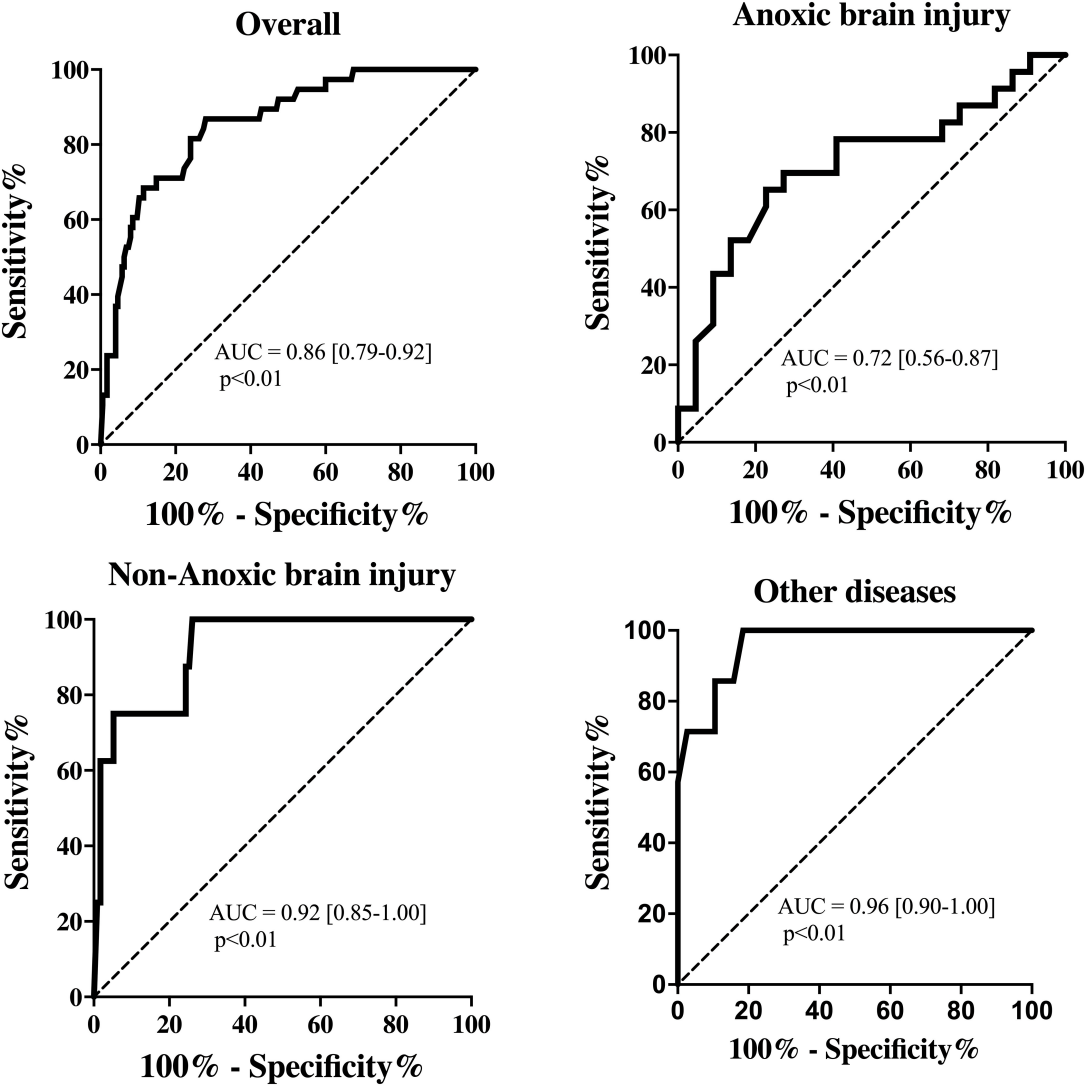
Receiver operating characteristic curve showing the accuracy of pupillary dilation velocity (DV) to predict unreactive EEG for all patients and among subgroups.

The EEG background was categorized as showing mild encephalopathy (*n* = 111; 52%), moderate encephalopathy (*n* = 65; 30%) or severe encephalopathy (*n* = 16; 8%); 19 patients (8%) had burst-suppression and 3 (1%) patients showed a suppressed background. Thirty-eight (18%) patients had an unreactive EEG. Seizures were observed in five patients (2%). The median NPi was 4.6 [4.3–4.8], and median CH was 31 [20–39] %, with a CV of 1.62 [0.95–2.44] mm/s and a DV of 0.64 [0.37–0.96] mm/s (Table 1 and Supplementary Table 2). There was a statistically significant difference for each of the parameters derived from automated pupillometry across the different EEG categories (Supplementary Table 4, Figure 1). Patients with an unreactive EEG had a significantly lower NPi, pupillary size, CH, MCV, CV and DV than patients with a reactive EEG and a higher latency time (Table 2 and Supplementary Table 5, Figure 2). The AUC for automated pupillometry-derived parameters showed they had good accuracy for predicting an unreactive EEG, in particular DV mean (AUC 0.86 [0.79–0.92]; *p* < 0.01); lower AUCs were observed for pupillary size, latency and NPi (Supplementary Table 6, Figure 3). Considering DV as the most accurate parameter from the AUC analysis, Youden's index identified a threshold of <0.50 mm/s to predict an unreactive EEG with a sensitivity of 87% and a specificity of 70%. High sensitivity (>95%) and high specificity (>95%) were obtained for DV values <0.85 mm/s and <0.20 mm/s, respectively (Supplementary Table 7).

**Table 2:** Characteristics of patients according to EEG reactivity (complete data are shown in Supplementary Table 5).

**Characteristic**	**Reactive EEG (*n* = 176)**	**Unreactive EEG (*n* = 38)**	***p* values**
Age, years	62 [50–72]	56 [48–70]	0.29
Men, *n* (%)	92 (52)	25 (66)	0.15
GCS	10 [6–14]	3 [3–3]	<0.001
Time from admission to test, days	2 [1–4]	1 [1–2]	0.01
**Comorbidities**			
COPD, *n* (%)	22 (13)	7 (18)	0.43
Heart disease, *n* (%)	61 (35)	15 (40)	0.58
Arterial hypertension, *n* (%)	78 (45)	16 (42)	0.86
Diabetes, *n* (%)	30 (17)	7 (18)	0.82
Liver cirrhosis, *n* (%)	12 (7)	3 (8)	0.74
Chronic renal disease, *n* (%)	24 (14)	9 (24)	0.14
Previous neurologic disease, *n* (%)	40 (23)	7 (18)	0.67
**Drugs during measure**			
Sedatives, *n* (%)	32 (18)	30 (79)	<0.001
Opioids, *n* (%)	51 (29)	27 (71)	<0.001
Analgosedation	119 (68)	6 (16)	<0.001
*No drugs, n (%)*	6 (3)	5 (13)	
*Sedatives, n (%)*	25 (14)	2 (5)	
*Opioids, n (%)*	26 (15)	25 (66)	
*Sedative and Opioids, n (%)*			
Vasopressors, *n* (%)	75 (43)	31 (82)	<0.001
Inotropes, *n* (%)	14 (8)	11 (29)	<0.001
**Pupillometry values**			
NPi Mean	4.7 [4.3–4.8]	4.3 [2.9–4.6]	<0.01
Size Mean, mm	3.46 [2.75–4.39]	2.53 [2.12–3.65]	<0.01
CH Mean, %	33 [23–40]	14 [6–26]	<0.01
CV Mean, mm/s	1.84 [1.11–2.55]	0.73 [0.45–1.20]	<0.01
MCV Mean, mm/s	3.00 [1.86–4.32]	1.10 [0.63–1.82]	<0.01
LAT Mean, sec	0.24 [0.22–0.27]	0.29 [0.25–0.33]	<0.01
DV Mean, mm/s	0.75 [0.46–1.01]	0.25 [0.14–0.42]	<0.01
**Outcome variables**			
ICU mortality, *n* (%)	41 (23)	29 (76)	<0.01
Hospital mortality, *n* (%)	55 (31)	32 (84)	<0.01

*Results given as count (%) or median [IQR]. GCS, Glasgow Coma Scale; COPD, Chronic Obstructive Pulmonary Disease; NPI, Neurologic Pupil Index; CH, Constriction Percentage; CV, Constriction Velocity; MCV, Maximum Constriction Velocity; LAT, Latency; DV, Dilation Velocity; ICU, Intensive Care Unit*.

### Predictors of Unreactive EEG

Patients with an unreactive EEG had a lower GCS (3 [3–3] vs. 10 [6–14]; *p* < 0.01), more frequently received sedative/opioids and had a significantly lower temperature, lower mean arterial pressure (MAP) and higher lactate blood level than other patients (Table 2 and Supplementary Table 5). In the multivariable logistic regression model, low DV, the combined use of sedative and analgesic drugs and high lactate concentrations were independently associated with unreactive EEG (Supplementary Table 8).

### Subgroup Analyses

Patients with anoxic brain injury had a lower GCS on the day of measurement and required sedatives more frequently, opioids and vasopressors than other patients; moreover, they had a lower temperature, higher lactate concentration, and were more frequently receiving mechanical ventilation than the two other subgroups. Patients with Non-anoxic brain injury were significantly younger and less frequently had chronic heart disease than the anoxic group. Patients with other diseases had a higher number of comorbidities (i.e., liver cirrhosis, chronic renal disease and immunosuppression), lower hemoglobin concentration, and more frequently required renal replacement therapy than patients in the other groups. Patients with anoxic brain injury had the highest ICU and hospital mortality rates (Table 1 and Supplementary Table 2).

Patients with anoxic brain injury more frequently had an unreactive EEG (*n* = 23; 51%), than patients with Non-anoxic brain injury (*n* = 8; 6%) or other diseases (*n* = 7; 16%). Pupillometry values were globally significantly different across the three subgroups of patients, even if in *post hoc* multiple comparisons, the difference was statistically significant only for some of them (Table 1 and Supplementary Table 2). In patients with anoxic brain injury, all automated pupillometry parameters had a lower predictive value for unreactive EEG than in other subgroups; this was particularly marked for DV (AUC 0.72 [0.56–0.87] for anoxic brain injury vs. 0.92 [0.85–1.00] for Non-anoxic brain injury and 0.96 [0.90–1.00] for other diseases; both *p* < 0.01 – Supplementary Table 6 and Supplementary Table 7; Figure 3).

### Pupillometry Variables, GCS and Mortality

DV (*r* = 0.67; *p* < 0.01) and MCV (*r* = 0.63; *p* < 0.01) correlated well with the GCS score, whereas the correlations for other parameters were moderate (Supplementary Table 9 and Supplementary Figure 1). The correlation between GCS and automated pupillometry parameters, in particular DV (*r* = 0.71; *p* < 0.01), MCV (*r* = 0.65; *p* < 0.01) and CV (*r* = 0.63; *p* < 0.01), was higher in patients with Non-anoxic brain injury than in patients in other subgroups (Supplementary Table 9). Non-survivors more frequently had an unreactive EEG compared to survivors [32 (37%) vs. 6 (5%), *p* < 0.01]. They also had a significantly lower pupillary size, CH, CV, MCV and DV than survivors, but a higher latency; NPi was similar between groups (Supplementary Table 10). Sensitivity analyses are reported in Supplementary Results 1, Supplementary Tables 11, 12.

## Discussion

In this study, a low DV was associated with an unreactive EEG, in particular in patients with Non-anoxic brain injury and other acute diseases and was an independent predictor of unreactive EEG in all patients. These results were confirmed when patients with mild encephalopathy or not receiving sedatives or opioids were excluded from the analysis. Automated pupillometry is therefore not only useful to assess brainstem impairment but also to identify patients with cortical dysfunction, which is characterized by an unreactive EEG background.

The potential relationship between cortical activity and pupillary function is related to the presence of multiple sympathetic and parasympathetic pathways, integrated at the midbrain level, which have connections to the neuronal activity of the gray matter, i.e., the locus coeruleus, colliculi and cingulate cortex (11, 12, 16). In a previous study, reduced pupillary constriction to light stimulation was also correlated to the severity of encephalopathy and the presence of an unreactive EEG (12); however, some automated pupillometry-derived parameters were not available and several confounders may have introduced important biases to the interpretation of these findings. In the present study, DV was the automated pupillometry-derived parameter that had the highest predictive value for an unreactive EEG. Pupil dilation, either to specific stimuli (e.g., pain, adrenergic drugs) or as a physiological phenomenon following recovery from pupillary constriction, depends on the contraction of the iris dilator muscle, which is controlled by the sympathetic nervous system (11). Locus coeruleus activation has been shown to evoke pupil dilation as well as to regulate arousal and, indirectly, cortical activation (17). Acute brain injuries or administration of sedative drugs, which are well-described causes of slow-frequency EEG background and severe encephalopathy, can affect locus coeruleus activity and therefore impair pupillary dilation. As such, the DV may represent the ideal variable derived from the analysis of pupillary function for further study to assess potential correlations with other EEG alterations, e.g., discontinuous background, attenuation and asymmetry, which are also important prognostic EEG findings in critically ill patients. The relatively low numbers of suppression and burst-suppression EEG traces limited further analyses (e.g., whether DV could further differentiate between these alterations and severe encephalopathy). Future studies should also assess whether changes in DV mirror changes in EEG background and reactivity in critically ill patients.

Few other studies have investigated the association of EEG findings with other neuro-monitoring tools. Serum neuron-specific enolase (NSE) levels and EEG abnormalities were strongly correlated in patients with anoxic brain injury (18), although this correlation was not always consistent or uniform (19). Although the presence of a continuous and reactive EEG background was almost universally associated with N20 cortical responses on somatosensory evoked potentials in cardiac arrest patients (20), no other correlations between these electrophysiological tests have been reported in other acute neurological diseases. In children with acute neurological disorders, EEG abnormalities were not correlated with alterations revealed by the cerebral CT-scan (21). Although an unreactive EEG does not have the same prognostic role as burst suppression or seizures in critically ill patients, it might still reflect the severity of the underlying disease or indicate over-sedation, and may therefore indicate the need for clinicians to order further diagnostic tests or adjust sedative and analgesic regimens in these patients. Importantly, our findings do not suggest that automated pupillometry could replace electroencephalography for monitoring critically ill patients; indeed, the main indications for electroencephalography are to monitor patients with status epilepticus, to detect Non-convulsive seizures in patients with unexplained alterations of consciousness, and to identify the occurrence of delayed cerebral ischemia in patients with SAH. No data are available on the association between these cerebral complications and automated pupillometry variables; moreover, assessing the depth of encephalopathy and/or detecting the presence of an unreactive background is not a common indication for electroencephalography. Above all, our data provide a physiological basis that links quantitative analysis of the pupils to cortical function, which might be useful for future clinical research and to help better implement the use of neuromonitoring in critically ill patients.

DV and other variables derived from automated pupillometry had limited predictive value for unreactive EEG in patients suffering from anoxic brain injury. Whether this observation is related to the type of brain injury, e.g., the brainstem is relatively resistant to anoxic events when compared to cortical areas, which could favor a dissociation in the abnormalities observed during electroencephalography and pupillary evaluation (22), or the more frequent use of sedatives and opioids in these patients remains unknown. Automated pupillometry would not be an adequate tool to predict the EEG background in these patients, although other derived parameters, such as NPi, have a high specificity to predict unfavorable neurological outcome in this patient population (13).

This study has several limitations. First, it was a single center study. Nevertheless, pupillary measurement has been described as having high inter-observer reproducibility (23). Second, although we considered the use of sedatives or analgesics in the multivariable analysis, we did not specifically calculate the cumulative dose of these drugs; in fact, sedatives may influence both EEG background, by reducing amplitude and frequency (24), EEG reactivity (25) and pupillary parameters, without specifically altering their relationship. Third, we analyzed a single automated pupillometry assessment and did not evaluate the reliability of repeated measurements for prediction of unreactive EEG. Fourth, a large proportion of eligible patients were not included because of the lack of an available operator, and this may have introduced a selection bias into the study findings. Fifth, we did not calculate a sample size to adequately test the study hypothesis, however a *post hoc* calculation showed a power of 95 and 97% for subgroups and reactivity analysis to assess the primary study hypothesis, respectively. Sixth, EEG reactivity is just a “measure” of brain injury and not a finding that requires a specific therapy; as such, whether a low DV may help in clinical practice remains to be established. Seventh, the definition of EEG reactivity can be debated, because even bursts can be stimulus-induced (i.e., a form of reactivity), although this might have a different significance in clinical practice. Eighth, although this is an easy and portable tool, AP is not yet accessible in many centers. Nineth, we did not specifically assess the association of AP variables with long-term neurological outcome, as this was beyond the scope of the study. Tenth, Glasgow Coma Scale was used to assess the level of consciousness, as in routine practice, while other scales, such as the Full Outline of UnResponsiveness (FOUR) score, might have provided more clinical information on the brainstem function. Eleventh, time from admission to test was heterogeneous and presence of TTM into the anoxic group may have leaded to a selection bias. Finally, brain imaging was not routinely evaluated and no association of potential structural cerebral lesions with EEG or pupillometry findings could not be analyzed.

## Conclusions

This study showed a significant association between automated pupillometry parameters, and in particular DV, and background reactivity of the EEG. Automated pupillometry can identify patients at risk of unreactive EEG, particularly among critically ill patients without anoxic brain injury.

## Data Availability Statement

The raw data supporting the conclusions of this article will be made available by the authors, without undue reservation.

## Ethics Statement

The studies involving human participants were reviewed and approved by Comité d'Ethique Hospitalo-Facultaire Erasme-ULB. Written informed consent for participation was not required for this study in accordance with the national legislation and the institutional requirements.

## Author Contributions

LP and FT conceived the study. LP, LF, FA, EG, and MT selected the population. LF reviewed all EEG recordings. LP, MT, NN, MM, and EM collected the data. LP, NG, and FT conducted the statistical analysis and wrote the first draft of the paper. BL, PS, JC, MO, J-LV, and NG revised the text for intellectual content. All authors contributed to the article and approved the submitted version.

## Conflict of Interest

FT and MO are scientific advisors for NeurOptics Inc. The remaining authors declare that the research was conducted in the absence of any commercial or financial relationships that could be construed as a potential conflict of interest.

## Publisher's Note

All claims expressed in this article are solely those of the authors and do not necessarily represent those of their affiliated organizations, or those of the publisher, the editors and the reviewers. Any product that may be evaluated in this article, or claim that may be made by its manufacturer, is not guaranteed or endorsed by the publisher.
