# Supplementary material for: Automated Pupillometry for Prediction of Electroencephalographic Reactivity in Critically Ill Patients: A Prospective Cohort Study
Source: Front Neurol. 2022 Mar 21;13:867603. doi: 10.3389/fneur.2022.867603 (PMC8977520; doi:10.3389/fneur.2022.867603)
Supplement: Supplementary file 1 [file Data_Sheet_1.docx]

***Supplemental Material***

***Automated Pupillometry for prediction of Electroencephalographic Reactivity***

***in Critically Ill Patients: a prospective cohort study***

*Lorenzo PELUSO^1^, MD; Lorenzo FERLINI^2^, MD; Marta TALAMONTI^1^, MD; Narcisse NDIEUGNOU DJANGANG^1^, MD; Elisa GOUVEA BOGOSSIAN^1^, MD; Marco MENOZZI^1^, MD; Filippo ANNONI^1^, MD; Elisabetta MACCHINI^1^, MD; Benjamin LEGROS^2^, MD, PhD; Paolo SEVERGNINI^3^, MD; Jacques CRETEUR^1^, MD, PhD; Mauro ODDO^4^, MD, Jean-Louis VINCENT^1^ MD, PhD, FCCM, Nicolas GASPARD^2,5^, MD, PhD; Fabio Silvio TACCONE^1^, MD, PhD*

^1^Department of Intensive Care

Erasme Hospital

Université libre de Bruxelles, Brussels, Belgium

^2^Department of Neurology

Cliniques Universitaires de Bruxelles Hopital Erasme

Route de Lennik, 808

1070, Brussels, Belgium

^3^Department of Biotechnology and Life Sciences

Insubria University

Cardiac Anesthesiology and Intensive Care - ASST Sette Laghi

Varese, Italy

^4^Critical Care Clinical Research Unit

Department of Intensive Care Medicine

CHUV-Lausanne University Hospital, Switzerland

^5^Department of Neurology

Yale University Medical School

15, York Street

06510 New Haven, CT, USA

**Supplemental Methods 1.**

STROBE Statement—Checklist of items that should be included in reports of ***cohort studies***

|  | Item No | Recommendation | Page |
| --- | --- | --- | --- |
| **Title and abstract** | 1 | (*a*) Indicate the study’s design with a commonly used term in the title or the abstract | 1 |
|  |  | (*b*) Provide in the abstract an informative and balanced summary of what was done and what was found | 2 |
| Introduction | | |  |
| Background/rationale | 2 | Explain the scientific background and rationale for the investigation being reported | 3 |
| Objectives | 3 | State specific objectives, including any prespecified hypotheses | 3 |
| Methods | | |  |
| Study design | 4 | Present key elements of study design early in the paper | 3,4 |
| Setting | 5 | Describe the setting, locations, and relevant dates, including periods of recruitment, exposure, follow-up, and data collection | 4 |
| Participants | 6 | (*a*) Give the eligibility criteria, and the sources and methods of selection of participants. Describe methods of follow-up | 4 |
|  |  | (*b*) For matched studies, give matching criteria and number of exposed and unexposed | NA |
| Variables | 7 | Clearly define all outcomes, exposures, predictors, potential confounders, and effect modifiers. Give diagnostic criteria, if applicable | 4 |
| Data sources/ measurement | 8* | For each variable of interest, give sources of data and details of methods of assessment (measurement). Describe comparability of assessment methods if there is more than one group | 4 |
| Bias | 9 | Describe any efforts to address potential sources of bias | 4 |
| Study size | 10 | Explain how the study size was arrived at | 4 |
| Quantitative variables | 11 | Explain how quantitative variables were handled in the analyses. If applicable, describe which groupings were chosen and why | 4,5 |
| Statistical methods | 12 | (*a*) Describe all statistical methods, including those used to control for confounding | 4,5 |
|  |  | (*b*) Describe any methods used to examine subgroups and interactions | 4,5 |
|  |  | (*c*) Explain how missing data were addressed | NA |
|  |  | (*d*) If applicable, explain how loss to follow-up was addressed | NA |
|  |  | (*e*) Describe any sensitivity analyses | 4,5 |
| Results | | |  |
| Participants | 13* | (a) Report numbers of individuals at each stage of study—eg numbers potentially eligible, examined for eligibility, confirmed eligible, included in the study, completing follow-up, and analysed | 5 |
|  |  | (b) Give reasons for non-participation at each stage | 5 |
|  |  | (c) Consider use of a flow diagram | NA |
| Descriptive data | 14* | (a) Give characteristics of study participants (eg demographic, clinical, social) and information on exposures and potential confounders | 5 |
|  |  | (b) Indicate number of participants with missing data for each variable of interest | 5 |
|  |  | (c) Summarise follow-up time (eg, average and total amount) |  |
| Outcome data | 15* | Report numbers of outcome events or summary measures over time | 5,6 |
| Main results | 16 | (*a*) Give unadjusted estimates and, if applicable, confounder-adjusted estimates and their precision (eg, 95% confidence interval). Make clear which confounders were adjusted for and why they were included | 6 |
|  |  | (*b*) Report category boundaries when continuous variables were categorized | 6 |
|  |  | (*c*) If relevant, consider translating estimates of relative risk into absolute risk for a meaningful time period | NA |
| Other analyses | 17 | Report other analyses done—eg analyses of subgroups and interactions, and sensitivity analyses | 6, Supplemental Mat. |
| Discussion | | |  |
| Key results | 18 | Summarise key results with reference to study objectives | 7 |
| Limitations | 19 | Discuss limitations of the study, taking into account sources of potential bias or imprecision. Discuss both direction and magnitude of any potential bias | 8 |
| Interpretation | 20 | Give a cautious overall interpretation of results considering objectives, limitations, multiplicity of analyses, results from similar studies, and other relevant evidence | 7,8 |
| Generalisability | 21 | Discuss the generalisability (external validity) of the study results | 8 |
| Other information | | |  |
| Funding | 22 | Give the source of funding and the role of the funders for the present study and, if applicable, for the original study on which the present article is based | 17 |

###

### **Supplemental Methods 2**

### Automated Pupillometry

The NPi®-200 pupillometer (Neuroptics, Laguna Hills, CA, USA) is a non-invasive device that uses an infrared camera with a calibrated light stimulation of fixed intensity (1000 Lux) and duration (3.2 seconds), enabling rapid and precise measurement (0.05 mm quantification limit) of the pupil size and of a series of dynamic pupillary variables (including the percentage of pupillary constriction [CH], CV, maximum CV [MCV], latency time, and dilation velocity [DV]). Based on the integration of these variables into an algorithm, the NPi®-200 pupillometer calculates the NPi, a scalar index with values between 0 and 5 (with a 0.1 decimal precision) [1]. NPi values < 3 are considered pathological [2]; the NPi is less influenced than other variables by external factors, such as the use of medication (e.g., opioids, analgesics) and the baseline pupil size [3]. For this study, in patients with NPi = 0, all other variables were considered equal to 0, except pupil size (given by the automated pupillometer) and latency (considered as missing). Pupillometry readings were taken from each patient on both eyes by the ICU medical staff, as part of standard of care. For this study, one operator (LP, MT or NND) performed the pupillary light reflex analysis, waiting for a minimum of 30 seconds between the two eyes for pupils to reach their baseline diameter after the stimulation. The timing of the pupillometry assessment was noted on the electroencephalograph screen. Results were given as the mean value for the two eyes; the mean worst value for the two eyes was also analyzed and reported.

**Supplemental Results 1.**

*Sensitivity analysis*

Excluding patients with mild encephalopathy, the CH, CV, MCV, latency and DV were significantly different in patients with unreactive and reactive EEGs (Supplemental Table 11); The DV had an AUC of 0.77 ([0.68-0.87]; p<0.01) to predict an unreactive EEG, which was significantly higher in patients without anoxic brain injury than in patients with anoxic injury (AUC 0.86 [0.76-0.96]; p<0.01 vs. AUC 0.67 [0.49-0.84], p=0.09).
 When only patients receiving sedatives and/or opioids were considered, all variables derived from the automated pupillometry, except the pupillary size, were significantly different in patients with unreactive and reactive EEGs (Supplemental Table 12); DV had a AUC of 0.73 ([0.60-0.86]; p<0.01) to predict an unreactive EEG, which was significantly higher in non-anoxic than in anoxic patients (AUC 0.86 [0.69-1.00]; p<0.01 vs. AUC 0.64 [0.45-0.83], p=0.16).

**Supplemental Table 1.** Modified Synek Encephalopathy Grading Scale.

| **Encephalopathy severity** | **Amplitude and continuity** | **Reactivity** | **Posterior dominant rhythm (PDR)** |
| --- | --- | --- | --- |
| Mild | >10uV | Present | Present |
| Moderate | >10uV | Present | Absent |
| Severe | 0-50% of  activity < 10uV | Absent | Absent |
| Burst suppression | 51%-99% of activity < 10uV | Absent | Absent |
| Suppression | No activity > 10uV | Absent | Absent |
|  |  |  |  |

**Supplemental Table 2.** Characteristics of the study population, according to diagnostic subgroup

|  | **Overall**  (n=214) | **Anoxic Brain Injury** (n=45) | **Non-Anoxic Brain Injury**  (n=124) | **Other Diseases**  (n=45) |
| --- | --- | --- | --- | --- |
| Age, years | 60 [50-72] | 68 [57-74]^a^ | 57 [48-70]^b^ | 66 [55-73]^a,b^ * |
| Men, n (%) | 117 (55) | 34 (76)^a^ | 58 (47)^b^ | 25 (56)^a,b^ * |
| GCS, n | 9 [3-14] | 3 [3-4]^a^ | 10 [7-14]^b^ | 10 [5-14]^b^ * |
|  |  |  |  |  |
| **Comorbidities** |  |  |  |  |
| COPD, n (%) | 29 (14) | 6 (14) | 15 (12) | 8 (18) |
| Asthma, n (%) | 5 (2) | 1 (2) | 4 (3) | - |
| Heart disease, n (%) | 76 (35) | 25 (57)^a^ | 27 (22)^b^ | 24 (53)^a^ * |
| Arterial hypertension, n (%) | 94 (44) | 22 (50) | 58 (47) | 14 (31) |
| Dyslipidemia, n (%) | 40 (19) | 12 (27) | 20 (16) | 8 (18) |
| Diabetes, n (%) | 37 (17) | 8 (18) | 20 (16) | 9 (20) |
| Liver cirrhosis, n (%) | 15 (7) | 1 (2)^a^ | 4 (3)^a^ | 10 (23)^b^ * |
| Immunosuppression, n (%) | 12 (6) | 1 (2)^a^ | 3 (2)^a^ | 8 (18)^b^ * |
| Chronic renal disease, n (%) | 33 (15) | 11 (25)^a^ | 9 (7)^b^ | 13 (29)^a^ * |
| Previous neurologic disease, n (%) | 47 (22) | 8 (18) | 27 (22) | 12 (27) |
|  |  |  |  |  |
| **Drugs during measurement** |  |  |  |  |
| Sedatives, n (%)  *Propofol, n (%)* | 62 (29)^+^  41 (19)^+^ | 34 (76)^a^  22 (49)^a^ | 20 (16)^b+^  13 (11)^b^ | 8 (18)^b+^ *  6 (13)^b^ * |
| *Midazolam, n (%)* | 19 (9) | 12 (27)^a^ | 4 (3)^b^ | 3 (7)^b+^ * |
| *Thiopenthal, n (%)* | 3 (1) | - | 3 (2) | - |
| *Ketamine, n (%)* | 1 (1)^+^ | - | 1 (1)^+^ | - |
| Opioids, n (%)  *Sufentanil, n (%)* | 78 (37)  53 (25) | 38 (84)^a^  30 (67)^a^ | 32 (26)^b^  16 (13)^b^ | 8 (18)^b^ *  7 (16)^b^ * |
| *Morphine, n (%)* | 25 (12) | 8 (18)^a^ | 16 (13)^a,b^ | 1 (2)^b^ * |
| Analgosedation  *No Drugs, n (%)*  *Sedatives, n (%)*  *Opioids, n (%)*  *Sedative and Opioids, n (%)*  Clonidine, n (%) | 125 (58)  11 (5)  27 (13)  51 (24)  11 (5) | 4 (9)^a^  3 (7)^a^  7 (15)^a^  31 (69)^a^  1 (2) | 86 (69)^b^  6 (5)^a^  18 (15)^a^  14 (11)^b^  7 (6) | 35 (79)^b^ *  2 (4)^a^  2 (4)^a^  6 (13)^b^  3 (7) |
| Antiepileptic drugs, n (%) | 99 (46) | 7 (16)^a^ | 84 (67)^b^ | 8 (18)^a^ * |
| NMBAs, n (%) | 23 (11) | 16 (36)^a^ | 5 (4)^b^ | 2 (4)^b^ * |
| Vasopressors, n (%) | 106 (50) | 37 (82)^a^ | 44 (36)^b^ | 25 (56)^b^ * |
| Inotropes, n (%) | 25 (12) | 18 (40)^a^ | 2 (2)^b^ | 5 (11)^c^ * |
| **Physiological variables during measure** |  |  |  |  |
| Mean arterial pressure, mmHg | 88 [79-104] | 78 [71-83]^a^ | 96 [86-109]^b^ | 81 [74-96]^a^ * |
| Temperature, °C | 36.3 [36.2-37.2] | 35.3 [33.3-36.9]^a^ | 37.0 [36.5-37.3]^b^ | 36.7 [36.3-37.2]^b^ * |
| Blood Lactate, mmol/L | 1.1 [0.8-1.7] | 2.1 [1.1-4.0]^a^ | 1.0 [0.7-1.4]^b^ | 1.4 [1.0-2.0]^a^ * |
| Hemoglobin, g/L | 11.0 [8.8-12.7] | 11.2 [9.0-12.9]^a^ | 11.2 [9.7-12.9]^a^ | 9.1 [7.9-11.6]^b^ * |
|  |  |  |  |  |
| **Life Supports** |  |  |  |  |
| Mechanical ventilation, n (%) | 117 (55) | 45 (100)^a^ | 52 (42)^b^ | 20 (44)^b^ * |
| ECMO, n (%) | 5 (2) | 2 (4) | 1 (1) | 2 (4) |
| RRT, n (%) | 18 (8) | 6 (13)^a^ | 2 (2)^b^ | 10 (22)^a^ * |
|  |  |  |  |  |
| **Pupillometry values** |  |  |  |  |
| NPi Mean | 4.6 [4.3-4.8] | 4.6 [4.3-4.8]^a,b^ | 4.6 [4.1-4.8]^a^ | 4.7 [4.4-4.8]^b^ * |
| NPi Worst | 4.5 [4.1-4.7] | 4.6 [4.2-4.7]^a,b^ | 4.5 [3.7-4.7]^a^ | 4.7 [4.3-4.8]^b^ * |
| Size Mean, mm | 3.37 [2.45-4.35] | 2.40 [2.04-3.22]^a^ | 3.64 [2.90-4.60]^b^ | 3.30 [2.39-4.22]^b^ * |
| Size Worst, mm | 3.08 [2.32-4.08] | 2.33 [1.93-3.05]^a^ | 3.32 [2.49-4.34]^b^ | 3.15 [2.26-3.98]^b^ * |
| CH Mean, % | 31 [20-39] | 21 [14-31]^a^ | 33 [21-40]^b^ | 34 [26-41]^b^ * |
| CH Worst, % | 29 [18-37] | 19 [12-29]^a^ | 29 [18-39]^b^ | 33 [24-40]^b^ * |
| CV Mean, mm/s | 1.62 [0.95-2.44] | 0.98 [0.65-1.33]^a^ | 1.91 [1.28-2.62]^b^ | 1.86 [1.03-2.39]^b^ * |
| CV Worst, mm/s | 1.46 [0.82-2.26] | 0.86 [0.54-1.14]^a^ | 1.75 [0.97-2.40]^b^ | 1.74 [0.88-2.23]^b^ * |
| MCV Mean, mm/s | 2.63 [1.50-4.06] | 1.46 [1.02-2.21]^a^ | 3.06 [1.98-4.48]^b^ | 3.06 [1.56-4.08]^b^ * |
| MCV Worst, mm/s | 2.24 [1.31-3.63] | 1.32 [0.88-1.97]^a^ | 2.77 [1.64-3.97]^b^ | 2.82 [1.42-3.82]^b^* |
| LAT Mean, sec | 0.25 [0.22-0.27] | 0.27 [0.24-0.30]^a^ | 0.23 [0.22-0.27]^b^ | 0.26 [0.23-0.29]^a^ * |
| LAT Worst, sec | 0.27 [0.23-0.30] | 0.27 [0.27-0.33]^a^ | 0.23 [0.23-0.27]^b^ | 0.27 [0.23-0.30]^a^ * |
| DV Mean, mm/s | 0.64 [0.37-0.96] | 0.37 [0.26-0.67]^a^ | 0.73 [0.42-1.01]^b^ | 0.79 [0.47-1.02]^b^ * |
| DV Worst, mm/s | 0.56 [0.30-0.87] | 0.33 [0.19-0.60]^a^ | 0.63 [0.35-0.90]^b^ | 0.74 [0.38-0.95]^b^ * |
|  |  |  |  |  |
| **EEG Features** |  |  |  |  |
| Background categories  *Mild encephalopathy*  *Moderate encephalopathy*  *Severe encephalopathy*  *Burst suppression*  *Suppression* | 111 (52)  65 (30)  16 (8)  19 (9)  3 (1) | 7 (16)^a^  15 (33)^a^  9 (20)^a^  12 (27)^a^  2 (4)^a^ | 80 (65)^b^  36 (29)^a^  3 (2)^b^  5 (4)^b^  0 (0)^a^ | *  24 (53)^b^  14 (31)^a^  4 (9)^a,b^  2 (5)^b^  1 (2)^a^ |
| Unreactive EEG, n (%) | 38 (18) | 23 (51)^a^ | 8 (6)^b^ | 7 (16)^b^ * |
| Seizures, n (%) | 5 (2) | - | 3 (3) | 2 (5) |
|  |  |  |  |  |
| **Outcome Variables** |  |  |  |  |
| ICU stay, days | 8 [4-16] | 6 [4-11]^a^ | 10 [6-18]^b^ | 6 [4-12]^a,b^ * |
| ICU mortality, n (%) | 70 (33) | 28 (62)^a^ | 29 (23)^b^ | 13 (29)^b^ * |
| Hospital mortality, n (%) | 87 (41) | 32 (71)^a^ | 36 (29)^b^ | 19 (43)^b^ * |

Results given as count (%) or median [IQR]. GCS = Glasgow Coma Scale; COPD = Chronic Obstructive Pulmonary Disease; NMBAs = Neuromuscular Blocking Agents; ECMO = Extracorporeal Membrane Oxygenation; RRT = Renal Replacement Therapy; NPi = Neurologic Pupil Index; CH = Constriction Percentage; CV = Constriction Velocity; MCV = Maximum Constriction Velocity; LAT = Latency; DV = Dilation Velocity; ICU = Intensive Care Unit

*= p < 0.05. Pairwise comparison calculated using Bonferroni correction and are expressed with superscript letters (equal letters indicate no difference among subgroups). ^+^= overlap of sedatives (propofol + midazolam and midazolam + ketamine)

**Supplemental Table 3.** Diagnosis on admission

| **Diagnose of Admission** | **Overall** |
| --- | --- |
|  | (n = 214) |
| **Anoxic Brain Injury Patients** | 45 (21) |
|  |  |
| **Non-Anoxic Brain Injury Patients** | 124 (58) |
| *Subarachnoid Hemorrhage* | 49 (23) |
| *Meningoencephalitis* | 2 (1) |
| *Ischemic Stroke* | 8 (4) |
| *Traumatic Brain Injury* | 22 (10) |
| *Intracranial Hemorrhage* | 24 (11) |
| *Seizure or epilepsy-related disorder* | 13 (6) |
| *Brain Neoplasm* | 2 (1) |
| *Hydrocephalus* | 3 (1) |
| *PRES* | 1 (1) |
|  |  |
| **Others Diseases Patients** | 45 (21) |
| *Sepsis or Septic Shock* | 26 (12) |
| *Liver Encephalopathy* | 8 (4) |
| *Drug Intoxication* | 3 (1) |
| *Hypoglycemia* | 1 (1) |
| *Other metabolic causes* | 7 (3) |

Data are expressed as count (percentage) PRES = Posterior Reversible Encephalopathy Syndrome

**Supplemental Table 4.** Pupillometry values according to EEG background categories.

|  | **Mild Encephalopathy**  **(n=111)** | **Moderate Encephalopathy**  **(n=65)** | **Severe Encephalopathy**  **(n=16)** | **Burst-Suppression**  **(n=19)** | **Suppressed Background**  **(n=3)** | ***p values*** |
| --- | --- | --- | --- | --- | --- | --- |
| NPi Mean | 4.7 [4.4-4.8] | 4.6 [4.0-4.8] | 4.4 [3.4-4.6] | 4.3 [3.0-4.8] | 4.2 [2.1-4.4] | 0.02 |
| NPi Worst | 4.6 [4.2-4.7] | 4.5 [3.6-4.7] | 4.2 [3.0-4.5] | 4.2 [2.8-4.7] | 4.0 [2.0-4.3] | 0.02 |
| Size Mean, mm | 3.75 [2.92-4.61] | 3.21 [2.26-4.24] | 2.66 [2.15-3.99] | 2.57 [2.10-3.53] | 2.46 [2.42-3.92] | < 0.01 |
| Size Worst, mm | 3.38 [2.59-4.28] | 2.99 [2.08-3.96] | 2.57 [2.08-3.36] | 2.44 [2.02-2.92] | 2.38 [2.36-3.72] | < 0.01 |
| CH Mean, % | 35 [28-42] | 26 [19-35] | 14 [8-27] | 12 [5-27] | 10 [5-14] | < 0.01 |
| CH Worst, % | 33 [25-40] | 24 [17-33] | 13 [5-24] | 11 [5-27] | 8 [4-13] | < 0.01 |
| CV Mean, mm/s | 2.12 [1.41-2.67] | 1.31 [0.83-2.04] | 0.73 [0.52-1.29] | 0.76 [0.41-1.17] | 0.63 [0.31-0.72] | < 0.01 |
| CV Worst, mm/s | 1.89 [1.31-2.44] | 1.11 [0.71-1.84] | 0.60 [0.28-1.01] | 0.58 [0.38-1.01] | 0.55 [0.28-0.59] | < 0.01 |
| MCV Mean, mm/s | 3.55 [2.31-4.61] | 2.18 [1.40-3.34] | 1.21 [0.78-2.22] | 1.16 [0.57-1.77] | 1.01 [0.51-1.02] | < 0.01 |
| MCV Worst, mm/s | 3.22 [2.05-4.20] | 1.83 [1.27-3.03] | 1.17 [0.55-1.71] | 0.88 [0.53-1.66] | 0.86 [0.43-0.90] | < 0.01 |
| LAT Mean, sec | 0.23 [0.22-0.27] | 0.25 [0.23-0.27] | 0.27 [0.25-0.31] | 0.30 [0.27-0.33] | 0.33 [0.28-0.37] | < 0.01 |
| LAT Worst, sec | 0.23 [0.23-0.27] | 0.27 [0.23-0.30] | 0.30 [0.27-0.33] | 0.30 [0.27-0.33] | 0.35 [0.33-0.37] | < 0.01 |
| DV Mean, mm/s | 0.85 [0.56-1.06] | 0.51 [0.36-0.79] | 0.23 [0.15-0.42] | 0.27 [0.11-0.40] | 0.18 [0.09-0.25] | < 0.01 |
| DV Worst, mm/s | 0.79 [0.45-0.97] | 0.46 [0.32-0.74] | 0.17 [0.10-0.24] | 0.23 [0.08-0.36] | 0.17 [0.09-0.23] | < 0.01 |

Results given as median [25th to 75th percentiles]

NPi = Neurologic Pupil Index; CH = Constriction Percentage; CV = Constriction Velocity; MCV = Maximum Constriction Velocity; LAT = Latency; DV = Dilation Velocity;

**Supplemental Table 5.** Characteristics of patients according to EEG reactivity

|  | **Reactive EEG**  (n=176) | **Unreactive EEG**  (n=38) | *p values* |  |
| --- | --- | --- | --- | --- |
| Age, years | 62 [50-72] | 56 [48-70] | 0.29 |  |
| Men, n (%) | 92 (52) | 25 (66) | 0.15 |  |
| GCS | 10 [6-14] | 3 [3-3] | <0.001 |  |
| Diagnosis on admission |  |  | <0.001 |  |
| *Anoxic Brain Injury, n (%)* | 22 (13) | 23 (61) |  |  |
| *Non-Anoxic Brain Injury, n (%)* | 116 (66) | 8 (21) |  |  |
| *Other Diseases, n (%)* | 38 (22) | 7 (18) |  |  |
|  |  |  |  |  |
| **Comorbidities** |  |  |  |  |
| COPD, n (%) | 22 (13) | 7 (18) | 0.43 |  |
| Asthma, n (%) | 5 (3) | - | 0.59 |  |
| Heart disease, n (%) | 61 (35) | 15 (40) | 0.58 |  |
| Arterial hypertension, n (%) | 78 (45) | 16 (42) | 0.86 |  |
| Dyslipidemia, n (%) | 34 (19) | 6 (16) | 0.82 |  |
| Diabetes, n (%) | 30 (17) | 7 (18) | 0.82 |  |
| Liver cirrhosis, n (%) | 12 (7) | 3 (8) | 0.74 |  |
| Immunosuppression, n (%) | 9 (5) | 3 (8) | 0.45 |  |
| Chronic renal disease, n (%) | 24 (14) | 9 (24) | 0.14 |  |
| Previous neurologic disease, n (%) | 40 (23) | 7 (18) | 0.67 |  |
|  |  |  |  |  |
| **Drugs during measure** |  |  |  |  |
| Sedatives, n (%)  *Propofol, n (%)*  *Midazolam, n (%)*  *Thiopenthal, n (%)*  *Ketamine, n (%)* | 32 (18)  22 (13)  9 (5)  1 (1)  - | 30 (79)  19 (50)  10 (26)  2 (5)  1 (3) | <0.001  <0.001  <0.001  0.08  0.18 |  |
| Opioids, n (%)  *Sufentanil, n (%)*  *Morphine, n (%)* | 51 (29)  33 (19)  18 (10) | 27 (71)  20 (53)  7 (18) | <0.001  <0.001  0.17 |  |
| Analgosedation  *No drugs, n (%)*  *Sedatives, n (%)*  *Opioids, n (%)*  *Sedative and Opioids, n (%)* | 119 (68)  6 (3)  25 (14)  26 (15) | 6 (16)  5 (13)  2 (5)  25 (66) | <0.001 |  |
| Clonidine, n (%) | 11 (6) | - | 0.21 |  |
| Antiepileptic drugs, n (%) | 83 (47) | 16 (42) | 0.60 |  |
| NMBAs, n (%) | 10 (6) | 13 (34) | <0.001 |  |
| Vasopressors, n (%) | 75 (43) | 31 (82) | <0.001 |  |
| Inotropes, n (%) | 14 (8) | 11 (29) | <0.001 |  |
|  |  |  |  |  |
| **Physiological variables during measure** |  |  |  |  |
| Mean arterial pressure, mmHg | 90 [80-105] | 80 [72-101] | 0.01 |  |
| Temperature, °C | 36.9 [36.5-37.3] | 36.2 [33.4-37.0] | <0.001 |  |
| Blood Lactate, mmol/L | 1.0 [0.8-1.5] | 3.1 [1.2-4.7] | <0.001 |  |
| Hemoglobin, g/L | 11 [8.9-12.7] | 10.9 [8.8-13.4] | 0.69 |  |
|  |  |  |  |  |
| **Life support** |  |  |  |  |
| Mechanical ventilation, n (%) | 80 (46) | 37 (97) | <0.001 |  |
| ECMO, n (%) | 3 (2) | 2 (5) | 0.22 |  |
| RRT, n (%) | 10 (6) | 8 (21) | 0.01 |  |
|  |  |  |  |  |
| **Pupillometry values** |  |  |  |  |
| NPi Mean | 4.7 [4.3-4.8] | 4.3 [2.9-4.6] | <0.01 |  |
| NPi Worst | 4.6 [4.2-4.7] | 4.2 [2.5-4.6] | <0.01 |  |
| Size Mean, mm | 3.46 [2.75-4.39] | 2.53 [2.12-3.65] | <0.01 |  |
| Size Worst, mm | 3.23 [2.38-4.16] | 2.45 [2.08-3.25] | <0.01 |  |
| CH Mean, % | 33 [23-40] | 14 [6-26] | <0.01 |  |
| CH Worst, % | 30 [19-39] | 12 [5-24] | <0.01 |  |
| CV Mean, mm/s | 1.84 [1.11-2.55] | 0.73 [0.45-1.20] | <0.01 |  |
| CV Worst, mm/s | 1.69 [0.95-2.33] | 0.58 [0.33-0.99] | <0.01 |  |
| MCV Mean, mm/s | 3.00 [1.86-4.32] | 1.10 [0.63-1.82] | <0.01 |  |
| MCV Worst, mm/s | 2.63 [1.54-3.86] | 0.91 [0.51-1.66] | <0.01 |  |
| LAT Mean, sec | 0.24 [0.22-0.27] | 0.29 [0.25.0.33] | <0.01 |  |
| LAT Worst, sec | 0.27 [0.23-0.27] | 0.30 [0.27-0.33] | <0.01 |  |
| DV Mean, mm/s | 0.75 [0.46-1.01] | 0.25 [0.14-0.42] | <0.01 |  |
| DV Worst, mm/s | 0.65 [0.36-0.92] | 0.17 [0.09-0.28] | <0.01 |  |
|  |  |  |  |  |
| **Outcome variables** |  |  |  |  |
| ICU stay, days | 9 [5-17] | 6 [4-11] | 0.01 |  |
| ICU mortality, n (%) | 41 (23) | 29 (76) | <0.01 |  |
| Hospital mortality, n (%) | 55 (31) | 32 (84) | <0.01 |  |

Results given as count (%) or median [IQR]. GCS = Glasgow Coma Scale; COPD = Chronic Obstructive Pulmonary Disease; NMBAs = Neuromuscular Blocking Agents; ECMO = Extracorporeal Membrane Oxygenation; RRT = Renal Replacement Therapy; NPI = Neurologic Pupil Index; CH = Constriction Percentage; CV = Constriction Velocity; MCV = Maximum Constriction Velocity; LAT = Latency; DV = Dilation Velocity; ICU = Intensive Care Unit

**Supplemental Table 6.** Areas under the receiver operating characteristics curves (AUC) for prediction of unreactive EEG according to primary diagnosis

|  | **AUC**  **Overall** | **AUC**  **Anoxic brain Injury** | **AUC**  **Non-Anoxic brain injury** | **AUC**  **Other Diseases** |
| --- | --- | --- | --- | --- |
| NPi Mean | 0.66 [0.56 - 0.76] | 0.67 [0.50 - 0.83] | 0.81 [0.65 – 0.97] | 0.74 [0.49 – 0.99] |
| NPi Worst | 0.66 [0.55 - 0.77] | 0.67 [0.50 – 0.83] | 0.78 [0.61 – 0.95] | 0.76 [0.52 – 1.00] |
| Size Mean | 0.76 [0.55 - 0.76] | 0.50 [0.32 – 0.67] | 0.64 [0.44 - 0.84] | 0.53 [0.27 – 0.78] |
| Size Worst | 0.65 [0.55 - 0.74] | 0.47 [0.29 – 0.64] | 0.63 [0.43 – 0.82] | 0.61 [0.37 – 0.84] |
| CH Mean | 0.82 [0.74 – 0.89] | 0.68 [0.52 – 0.84] | 0.87 [0.72 – 1.00] | 0.91 [0.81 – 1.00] |
| CH Worst | 0.80 [0.72 – 0.87] | 0.67 [0.51 – 0.83] | 0.85 [0.70 – 1.00] | 0.92 [0.83 – 1.00] |
| CV Mean | 0.84 [0.77 – 0.90] | 0.66 [0.50 – 0.82] | 0.86 [0.70 – 1.00] | 0.92 [0.82 – 1.00] |
| CV Worst | 0.83 [0.77 – 0.90] | 0.68 [0.52 – 0.83] | 0.84 [0.65 – 1.00] | 0.94 [0.86 – 1.00] |
| MCV Mean | 0.84 [0.78 – 0.91] | 0.68 [0.52 – 0.84] | 0.87 [0.71 – 1.00] | 0.94 [0.85 – 1.00] |
| MCV Worst | 0.83 [0.76 – 0.90] | 0.66 [0.51 – 0.83] | 0.86 [0.69 – 1.00] | 0.96 [0.90 – 1.00] |
| LAT Mean | 0.78 [0.70 – 0.87] | 0.75 [0.60 – 0.89] | 0.63 [0.39 – 0.86] | 0.91 [0.83 – 1.00] |
| LAT Worst | 0.77 [0.69 – 0.86] | 0.73 [0.58 – 0.88] | 0.62 [0.36 – 0.88] | 0.91 [0.82 – 1.00] |
| DV Mean | 0.86 [0.79 – 0.92] | 0.72 [0.56 – 0.87] | 0.92 [0.85 – 1.00] | 0.96 [0.90 – 1.00] |
| DV Worst | 0.85 [0.79 – 0.92] | 0.74 [0.59 – 0.89] | 0.91 [0.83 – 1.00] | 1.00 [1.00 – 1.00] |

Results given as AUC [95% C.I.] NPI = Neurologic Pupil Index; CH = Constriction Percentage; CV = Constriction Velocity; MCV = Maximum Constriction Velocity; LAT = Latency; DV = Dilation Velocity.

**Supplemental Table 7.** Different thresholds and predictive values for unreactive EEG, according to subgroups.

|  | **OVERALL** | | | |
| --- | --- | --- | --- | --- |
| DV Value (mm/sec) | *Sensitivity*  *[95% CI]* | *Specificity*  *[95% CI]* | *Negative Predictive Value [95% CI]* | *Positive Predictive Value [95% CI]* |
| 0.20 | 45 [29-62] | 94 [90-97] | 89 [85-91] | 63 [46-77] |
| 0.50 | 87 [72-96] | 70 [62-76] | 96 [91-98] | 38 [33-45] |
| 0.85 | 97 [86-100] | 38 [31-46] | 99 [91-100] | 26 [23-28] |
|  | **ANOXIC BRAIN INJURY** | | | |
|  | *Sensitivity*  *[95% CI]* | *Specificity*  *[95% CI]* | *Negative Predictive Value [95% CI]* | *Positive Predictive Value [95% CI]* |
| 0.15 | 17 [5-39] | 95 [77-100] | 53 [47-58] | 80 [33-97] |
| 0.30 | 52 [31-73] | 82 [60-95] | 62 [51-72] | 75 [53-89] |
| 0.90 | 9 [1-29] | 96 [78-100] | 67 [16-95] | 52 [48-56] |
|  | **NON-ANOXIC BRAIN INJURY** | | | |
|  | *Sensitivity*  *[95% CI]* | *Specificity*  *[95% CI]* | *Negative Predictive Value [95% CI]* | *Positive Predictive Value [95% CI]* |
| 0.20 | 75 [35-97] | 93 [87-97] | 98 [94-99] | 43 [26-62] |
| 0.50 | 100 [63-100] | 73 [64-81] | 100 | 21 [16-26] |
|  | **OTHER DISEASES** | | | |
|  | *Sensitivity*  *[95% CI]* | *Specificity*  *[95% CI]* | *Negative Predictive Value [95% CI]* | *Positive Predictive Value [95% CI]* |
| 0.30 | 71 [29-96] | 97 [86-100] | 95 [85-98] | 83 [41-97] |
| 0.50 | 100 [59-100] | 76 [60-89] | 100 | 44 [31-58] |

The first value is calculated to obtain a specificity ≥ 95%, the second value is calculated with Youden’s index, the third value is calculated to obtain a sensitivity ≥ 95%. For not-anoxic brain injury and others diseases patients the value obtained with youden’s index was the same of the highest sensitivity.

**Supplemental Table 8.** Logistic regression analysis to predict unreactive EEG

|  | **UNIVARIATE** | | | **MULTIVARIABLE** | | |
| --- | --- | --- | --- | --- | --- | --- |
|  | **OR** | **95% CI** | **p value** | **OR** | **95% CI** | **p value** |
| **Mean Pupillary Dilation Velocity**  **Blood Lactate concentration** | 0.006  1.99 | 0.001 – 0.037  1.52 – 2.60 | < 0.01  < 0.01 | 0.02  1.82 | 0.002 – 0.163  1.33 – 2.49 | <0.01  <0.01 |
| **Analgo-sedation**  *No sedation*  *Sedatives*  *Analgesia*  *Sedatives and analgesia* | 0  16.53  1.59  19.07 | -  3.91 – 69.94  0.30 – 8.32  7.11 – 51.16 | -  <0.01  0.59  < 0.01 | 0  5.83  0.81  5.54 | -  0.99 – 34.40  0.11 – 6.00  1.72 – 17.83 | -  0.051  0.83  <0.01 |

Legend: OR = Odds Ratio, CI = Confidence Interval

Hosmer and Lemeshow goodness-of-fit= 0.83

**Supplemental Table 9.** Correlation indexes between the Glasgow Coma Scale (GCS) score on the day of pupillometry assessment and variables derived from automated pupillometry, according to the primary diagnosis.

|  | **Overall**  **(n = 214)** | **Anoxic brain injury**  **(n = 45)** | **Non-Anoxic brain injury**  **(n = 124)** | **Other diseases**  **(n = 45)** |
| --- | --- | --- | --- | --- |
| NPi Mean | r = 0.18  p < 0.01 | r = 0.11  p = 0.49 | r = 0.28  p < 0.01 | r = 0.25  p = 0.11 |
| NPi Worst | r = 0.17  p < 0.01 | r = 0.10  p = 0.53 | r = 0.27  p < 0.01 | r = 0.24  p = 0.13 |
| Size Mean | r = 0.42  p < 0.01 | r = 0.16  p = 0.29 | r = 0.41  p < 0.01 | r = 0.15  p = 0.35 |
| Size Worst | r = 0.43  p < 0.01 | r = 0.16  p = 0.28 | r = 0.43  p < 0.01 | r = 0.22  p = 0.17 |
| CH Mean | r = 0.58  p < 0.01 | r = 0.23  p = 0.12 | r = 0.58  p < 0.01 | r = 0.49  p < 0.01 |
| CH Worst | r = 0.56  p < 0.01 | r = 0.22  p = 0.16 | r = 0.57  p < 0.01 | r = 0.49  p < 0.01 |
| CV Mean | r = 0.62  p < 0.01 | r = 0.30  p = 0.04 | r = 0.63  p < 0.01 | r = 0.40  p < 0.01 |
| CV Worst | r = 0.61  p < 0.01 | r = 0.28  p = 0.07 | r = 0.61  p < 0.01 | r = 0.41  p < 0.01 |
| MCV Mean | r = 0.63  p < 0.01 | r = 0.27  p = 0.07 | r = 0.65  p < 0.01 | r = 0.42  p < 0.01 |
| MCV Worst | r = 0.62  p < 0.01 | r = 0.25  p = 0.11 | r = 0.64  p < 0.01 | r = 0.43  p < 0.01 |
| LAT Mean | r = -0.32  p < 0.01 | r = -0.10  p = 0.51 | r = -0.22  p = 0.02 | r = -0.36  p = 0.03 |
| LAT Worst | r = -0.30  p < 0.01 | r = -0.04  p = 0.81 | r = -0.20  p = 0.03 | r = -0.36  p = 0.02 |
| DV Mean | r = 0.67  p < 0.01 | r = 0.38  p = 0.01 | r = 0.71  p < 0.01 | r = 0.53  p < 0.01 |
| DV Worst | r = 0.62  p < 0.01 | r = 0.34  p = 0.02 | r = 0.66  p < 0.01 | r = 0.50  p < 0.01 |

Correlation indexes calculated using Pearson’s or Spearman’s test, as appropriate. Legend: NPi = Neurologic Pupil Index; CH = Constriction Percentage; CV = Constriction Velocity; MCV = Maximum Constriction Velocity; LAT = Latency; DV = Dilation Velocity

**Supplemental Table 10.** Pupillometry values and EEG features according to hospital mortality.

|  | **SURVIVORS**  **(n=127)** | **NON-SURVIVORS**  **(n=87)** | ***p values*** |
| --- | --- | --- | --- |
| **Pupillometry values** |  |  |  |
| NPi Mean | 4.7 [4.3-4.8] | 4.6 [3.9-4.8] | 0.15 |
| NPi Worst | 4.6 [4.2-4.7] | 4.5 [3.4-4.7] | 0.16 |
| Size Mean, mm | 3.60 [2.80-4.61] | 2.94 [2.28-3.90] | < 0.01 |
| Size Worst, mm | 3.38 [2.45-4.39] | 2.73 [2.12-3.45] | < 0.01 |
| CH Mean, % | 33 [25-41] | 23 [13-33] | < 0.01 |
| CH Worst, % | 31 [22-39] | 20 [10-32] | < 0.01 |
| CV Mean, mm/s | 2.01 [1.17-2.67] | 1.20 [0.71-1.62] | < 0.01 |
| CV Worst, mm/s | 1.84 [1.03-2.45] | 0.99 [0.57-1.46] | < 0.01 |
| MCV Mean, mm/s | 3.30 [2.01-4.51] | 1.82 [1.04-2.60] | < 0.01 |
| MCV Worst, mm/s | 2.95 [1.69-4.15] | 1.58 [0.88-2.24] | < 0.01 |
| LAT Mean, sec | 0.23 [0.22-0.27] | 0.27 [0.23-0.30] | < 0.01 |
| LAT Worst, sec | 0.23 [0.23-0.27] | 0.27 [0.23-0.32] | < 0.01 |
| DV Mean, mm/s | 0.82 [0.47-1.05] | 0.42 [0.25-0.71] | < 0.01 |
| DV Worst, mm/s | 0.77 [0.39-0.94] | 0.33 [0.17-0.66] | < 0.01 |
|  |  |  |  |
| **EEG Features** |  |  |  |
| Background categories  *Mild encephalopathy*  *Moderate encephalopathy*  *Severe encephalopathy*  *Burst suppression*  *Suppression* | 85 (67)  36 (28)  4 (3)  2 (2)  - | 26 (30)  29 (33)  12 (14)  17 (20)  3 (3) | < 0.01 |
| Unreactive, n (%) | 6 (5) | 32 (37) | < 0.01 |
| Seizures, n (%) | 2 (2) | 3 (4) | 0.40 |

Results given as median [25th to 75th percentiles] or counts (percentage)

NPi = Neurologic Pupil Index; CH = Constriction Percentage; CV = Constriction Velocity; MCV = Maximum Constriction Velocity; LAT = Latency; DV = Dilation Velocity;

**Supplemental Table 11.** Pupillometry values and EEG features according to reactivity, excluding patients with mild encephalopathy.

|  | **REACTIVE**  **(n=65)** | **UNREACTIVE**  **(n=38)** | ***p values*** |
| --- | --- | --- | --- |
| **Glasgow Coma Scale**  **Pupillometry values** | 6 [3-9] | 3 [3-3] | < 0.01 |
| NPi Mean | 4.6 [4.0-4.8] | 4.3 [2.9-4.6] | 0.03 |
| NPi Worst | 4.5 [3.6-4.7] | 4.2 [2.5-4.6] | 0.04 |
| Size Mean, mm | 3.21 [2.26-4.24] | 2.53 [2.12-3.65] | 0.14 |
| Size Worst, mm | 2.99 [2.08-3.96] | 2.45 [2.08-3.25] | 0.20 |
| CH Mean, % | 26 [19-35] | 14 [6-26] | < 0.01 |
| CH Worst, % | 24 [17-33] | 12 [5-24] | < 0.01 |
| CV Mean, mm/s | 1.31 [0.83-2.04] | 0.73 [0.45-1.20] | < 0.01 |
| CV Worst, mm/s | 1.11 [0.71-1.84] | 0.58 [0.33-0.99] | < 0.01 |
| MCV Mean, mm/s | 2.18 [1.40-3.34] | 1.10 [0.63-1.82] | < 0.01 |
| MCV Worst, mm/s | 1.83 [1.27-3.03] | 0.91 [0.51-1.66] | < 0.01 |
| LAT Mean, sec | 0.25 [0.23-0.27] | 0.29 [0.25.0.33] | < 0.01 |
| LAT Worst, sec | 0.27 [0.23-0.30] | 0.30 [0.27-0.33] | < 0.01 |
| DV Mean, mm/s | 0.51 [0.36-0.79] | 0.25 [0.14-0.42] | < 0.01 |
| DV Worst, mm/s | 0.46 [0.32-0.74] | 0.17 [0.09-0.28] | < 0.01 |
|  |  |  |  |
| **EEG Features** |  |  |  |
| Background categories  *Moderate encephalopathy*  *Severe encephalopathy*  *Burst suppression*  *Suppression* | 65 (100)  -  -  - | -  16 (42)  19 (50)  3 (8) | < 0.01 |
|  |  |  |  |
| Seizures, n (%) | - | 3 (8) | 0.05 |

Results given as median [25th to 75th percentiles] or counts (percentage)

GCS = Glasgow Coma Scale; NPi = Neurologic Pupil Index; CH = Constriction Percentage; CV = Constriction Velocity; MCV = Maximum Constriction Velocity; LAT = Latency; DV = Dilation Velocity.

**Supplemental Table 12.** Pupillometry values and EEG features according to reactivity, excluding patients without sedative medication

|  | **REACTIVE**  **(n=32)** | **UNREACTIVE**  **(n=30)** | ***p values*** |
| --- | --- | --- | --- |
| **Glasgow Coma Scale**  **Pupillometry values** | 3 [3-7] | 3 [3-3] | < 0.01 |
| NPi Mean | 4.7 [4.3-4.8] | 4.3 [2.7-4.7] | 0.03 |
| NPi Worst | 4.7 [4.3-4.8] | 4.2 [2.5-4.7] | 0.03 |
| Size Mean, mm | 2.28 [2.00-3.15] | 2.46 [2.12-3.65] | 0.20 |
| Size Worst, mm | 2.18 [1.91-2.96] | 2.41 [2.08-3.03] | 0.15 |
| CH Mean, % | 20 [17-26] | 13 [5-25] | 0.03 |
| CH Worst, % | 19 [15-24] | 11 [4-24] | 0.07 |
| CV Mean, mm/s | 1.00 [0.72-1.35] | 0.67 [0.37-1.01] | < 0.01 |
| CV Worst, mm/s | 0.89 [0.62-1.14] | 0.54 [0.25-0.91] | 0.01 |
| MCV Mean, mm/s | 1.54 [1.20-2.13] | 1.03 [0.52-1.49] | < 0.01 |
| MCV Worst, mm/s | 1.36 [1.03-1.70] | 0.87 [0.51-1.38] | 0.01 |
| LAT Mean, sec | 0.25 [0.22-0.29] | 0.30 [0.26-0.33] | < 0.01 |
| LAT Worst, sec | 0.27 [0.23-0.30] | 0.33 [0.27-0.33] | < 0.01 |
| DV Mean, mm/s | 0.37 [0.33-0.49] | 0.24 [0.08-0.41] | < 0.01 |
| DV Worst, mm/s | 0.35 [0.28-0.48] | 0.19 [0.06-0.29] | < 0.01 |
|  |  |  |  |
| **EEG Features** |  |  |  |
| Background categories  *Mild encephalopathy*  *Moderate encephalopathy*  *Severe encephalopathy*  *Burst suppression*  *Suppression* | 7 (22)  25 (78)  -  -  - | -  -  9 (30)  18 (60)  3 (10) | < 0.01 |
|  |  |  |  |
| Seizures, n (%) | - | 2 (7) | 0.49 |

Results given as median [25th to 75th percentiles] or counts (percentage)

GCS = Glasgow Coma Scale; NPi = Neurologic Pupil Index; CH = Constriction Percentage; CV = Constriction Velocity; MCV = Maximum Constriction Velocity; LAT = Latency; DV = Dilation Velocity.

**Supplemental Figure 1.** Correlation of Glasgow Coma Score with Dilation Velocity (DV) and Maximum Constriction Velocity (MCV) with Glasgow Coma Scale (GCS).

**
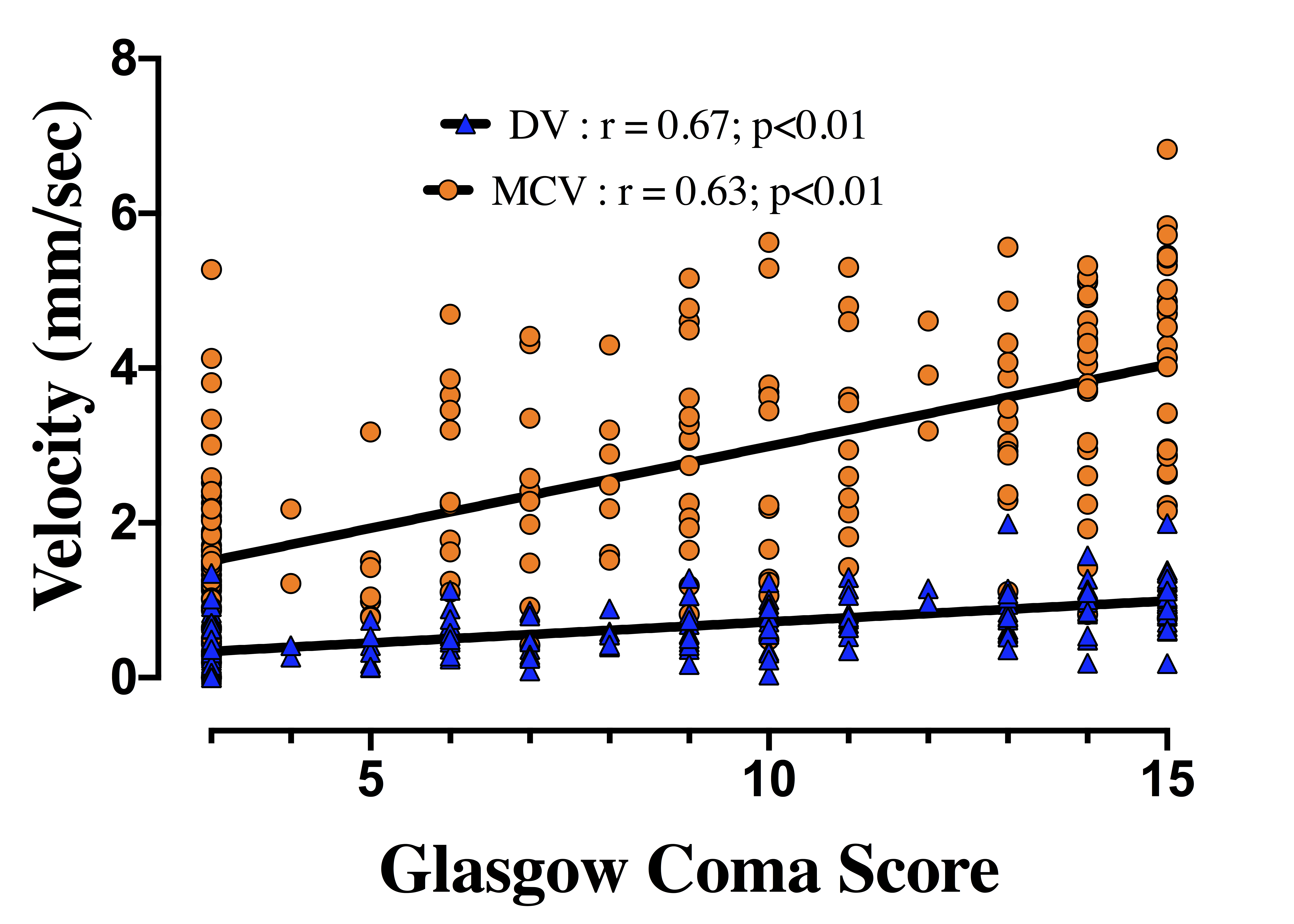
**

**REFERENCES**

1. Quispe Cornejo A, Fernandes Vilarinho CS, Crippa IA, Peluso L, Calabro L, Vincent JL, Creteur J, Taccone FS: **The use of automated pupillometry to assess cerebral autoregulation: a retrospective study**. *J Intensive Care* 2020, **8**:57.

2. Miroz JP, Ben-Hamouda N, Bernini A, Romagnosi F, Bongiovanni F, Roumy A, Kirsch M, Liaudet L, Eckert P, Oddo M: **Neurological Pupil index for Early Prognostication After Venoarterial Extracorporeal Membrane Oxygenation**. *Chest* 2020, **157**(5):1167-1174.

3. Olson DM, Fishel M: **The Use of Automated Pupillometry in Critical Care**. *Crit Care Nurs Clin North Am* 2016, **28**(1):101-107.
